# Microplastics in Sediment and Surface Water of West Dongting Lake and South Dongting Lake: Abundance, Source and Composition

**DOI:** 10.3390/ijerph15102164

**Published:** 2018-10-01

**Authors:** Changbo Jiang, Lingshi Yin, Xiaofeng Wen, Chunyan Du, Lixue Wu, Yuannan Long, Yizhuang Liu, Yuan Ma, Qide Yin, Zhenyu Zhou, Hemin Pan

**Affiliations:** 1 School of Hydraulic Engineering, Changsha University of Science & Technology, Changsha 410114, China; yin@csust.edu.cn (L.Y.); cydu@csust.edu.cn (C.D.); wlx1709@stu.csust.edu.cn (L.W.); lynzhb@163.com (Y.Long); carl-lyz@foxmail.com (Y.Liu); mayuan1026@gmail.com (Y.M.); 2453690532@stu.csust.edu.cn (Z.Z.); wfdphm@163.com (H.P.); 2 Key Laboratory of Dongting Lake Aquatic Eco-Environmental Control and Restoration of Hunan Province, Changsha 410114, China; 3 College of Environmental Science and Engineering, Ministry of Education, Hunan University and Key Laboratory of Environmental Biology and Pollution Control (Hunan University), Changsha 410082, China; wenxf0105@hnu.edu.cn; 4 School of Chemistry and Biological Engineering, Changsha University of Science & Technology, Changsha 410114, China; yinls@stu.csust.edu.cn

**Keywords:** microplastic, Dongting Lake, surface water, sediment

## Abstract

Microplastic pollution was investigated in sediment and surface water in West Dongting Lake and South Dongting Lake for the first time. The abundance of microplastics ranged from 616.67 to 2216.67 items/m^3^ and 716.67 to 2316.67 items/m^3^ in the lakeshore surface water of West Dongting Lake and South Dongting Lake, respectively. The highest levels of microplastic pollution were found in the lakes’ outlets. In the lake center sites of the West Dongting Lake and South Dongting Lake, the abundance of microplastics ranged from 433.33 to 1500 items/m^3^ and 366.67 to 1566.67 items/m^3^, respectively. Meanwhile, the study found that in lakeshore sediment of West Dongting Lake and South Dongting Lake, microplastic concentrations ranged from 320 to 480 items/m^3^ and 200–1150 items/m^3^. Polystyrene (PS) and polyethylene terephthalate (PET) were most common in the surface water and sediment samples, respectively. In addition, we suggest that the effects of polymer types in microplastics should be taken into account when considering abundance. This study can provide valuable points of reference to better understanding microplastic pollution in inland freshwater areas.

## 1. Introduction

Plastic is a macromolecular compound that is polymerized by adding or condensing a monomeric raw material. Worldwide plastic production reached 335 million tons in 2016 [1]. Plastics bring convenience to people’s lives; however, plastics products also lead to environmental problems. Plastics may not be decompose in the natural environment for tens to hundreds of years due to their stable physical and chemical structures [2,3]. Thus, the pollution of plastic waste from petrochemical production is a difficult problem worldwide. 

Plastic debris with a size <5 mm is defined as a microplastic [4]. Microplastics in the environment can be divided into two categories: primary microplastics and secondary microplastics [5,6]. Plastic particles with a size <5 mm at the time of production are called primary microplastics, while secondary microplastics are particles fragmented from larger plastic debris. As an emerging pollutant, microplastics have attracted much attention in recent years [7,8,9]. According to published studies, microplastics have spread all over the marine environment, including remote polar regions [10,11,12]. Microplastics in the marine environment can exert adverse effects on marine organisms; these effects include damage to energy intake, hormone secretion, growth rate, and reproductive capacity [13,14,15]. Notably, for some aquatic organisms, such as *Sciaenidae* and *Gymnocypris przewalskii*, the size of microplastics is very similar to the size of their food [16,17]. In fact, microplastics have been found in aquatic animals when the biotoxicity of microplastics has been studied by exposure tests [18,19]. These studies show that when microplastics are eaten by aquatic animals, they not only affect the animal that eats them, but they also accumulate in the food chain and eventually affect human beings and other top predators [20,21,22]. In addition, structure and size of microplastics provide breeding or attachment sites for microbes or pollutants [22].

Marine microplastic pollution has received particular attention, but inland freshwater areas also face the threat of microplastics. According to estimates of a published study, more than 95% of plastic waste remained in the terrestrial environment [23]. Although people keep large amounts of plastic waste in the soil through landfills and other forms of waste disposal, the microplastics that are transported to the fresh water system in a variety of ways still merit attention. Freshwater systems are important receptors of microplastic and transport networks to the ocean. High concentrations of microplastics have been detected in many rivers and lakes [17,24,25]. Levels of microplastic pollution in many freshwater areas are on par with or worse than marine waters or sediments [26]. Since humans have frequent contact with such areas, it is necessary to conduct microplastic pollution research, as microplastics also have been found in relatively remote and unfrequented areas [27,28].

China is the largest producer and consumer of plastics in the world. Dongting Lake, located in the northern Hunan Province (N 27°39′–29°51′, E 111°19′–113°34′), is the second largest freshwater lake in China. Its average depth is 6.4 m, and its volume is 1.67 billion m^3^. Dongting Lake covers an area of about 2625 km^2^ and is divided into three parts due the influence of lake reclamation and siltation. From upstream to downstream these are named West Dongting Lake, South Dongting Lake and East Dongting Lake.

Dongting Lake is an important agricultural base and tourist spot. It is also a large natural park and a genetic gene bank. There are 863 species of wetland plants, 164 bird species, and 114 fish species, including *Acipenser sinensis*, *Grus leucogeranus*, *Grus monacha*, and *Mergus squamatus*, as well as other important protected animals. In addition, the area has more than 11 million permanent residents, accounting for 20% of the population of Hunan Province. The four main rivers in Hunan Province (Xiangjiang River, Zishui River, Yuanjiang River, and Lishui River) all inflow to the West Dongting Lake and the South Dongting Lake. The abundant freshwater reserves and strong storage capacity of Dongting Lake are of great importance to the lake area and the whole middle and lower reaches of the Yangtze River. Until now, only microplastics from surface water in East Dongting Lake have been investigated [29]. However, as the other two important parts of Dongting Lake, West Dongting Lake and South Dongting Lake also merit further study, especially since the booming agriculture and tourism industry is already putting pressure on the environment of the research areas [30,31,32,33]. As of now, there has been no published study on microplastic pollution in West Dongting Lake and South Dongting Lake, and people know nothing about microplastic pollution in South Dongting Lake. Therefore, this study investigated microplastics in the surface water and lakeshore sediment of West Dongting Lake and South Dongting Lake with the purposes of: investigating microplastic pollution levels and distribution features and explaining the sources and composition of the microplastics.

## 2. Materials and Methods 

### 2.1. Sampling

**Figure 1:**
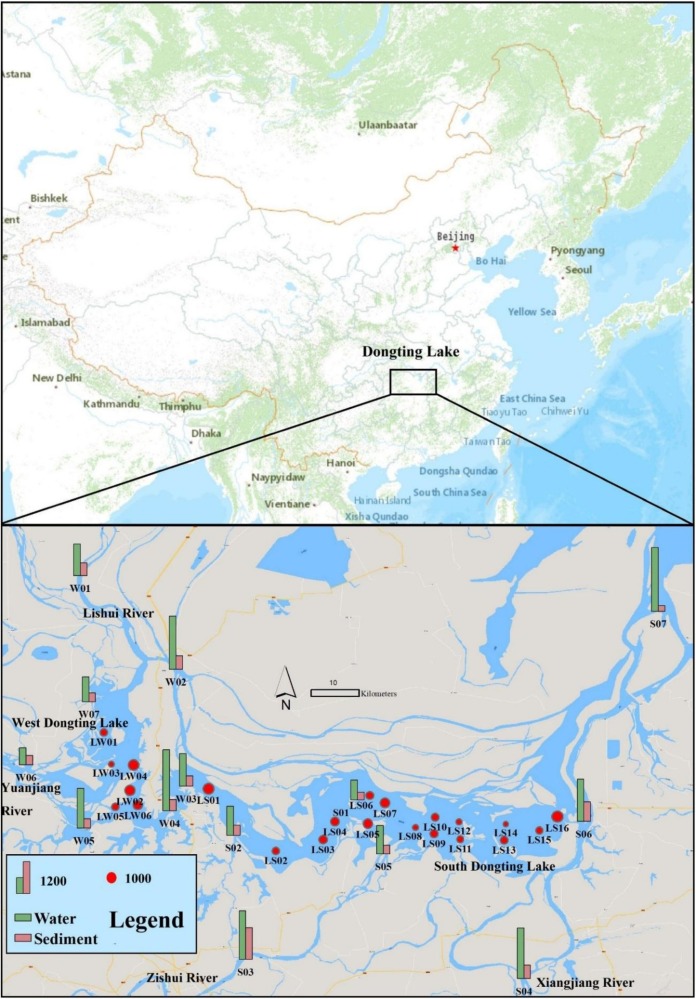
Geographic locations, sampling sites and microplastic abundance (The columns represent the lakeshore sites, and the pies represent the lake center sampling sites, and the units of microplastic concentration in water and sediment are items/m^3^ and items/kg, respectively).

The sampling was performed in April 2018. The geographic location and related information of the sampling sites are presented in Figure 1 and Table 1. The location of the sampling sites was determined using a Global Positioning System (GPS, GEOXT, Trimble, Sunnyvale, CA, USA). In this study, waterfront sampling sites are defined as ‘lakeshore sites’, and those far from the shore and near the center of the lake are defined as “lake center sites”. 

Fourteen lakeshore sites and 22 lake center sites were selected as sampling sites. On the basis of the control section settled by the environmental protection department of China, the representative shore points were selected as lakeshore sampling sites. The sampling sites in the lake center sites were equally distributed. Both surface water and sediment samples were collected at lakeshore sites, while only surface water samples were collected at lake center sites. 

Surface water and sediment samples were collected using a previously reported method with some modifications [11,34,35,36]. Briefly, in the sampling of surface water, thirty liter surface water samples (0–30 cm in depth) were collected by a pre-cleaned large flow sampler (KLL-S4, SEBA, Kaufbeuren, Germany). The collected water was filtered with a 45 μm stainless sieve. All of the solids on the sieve were rinsed carefully into a 1 L glass jar with deionized water; a 5% formalin solution was selected as preservative. Three duplicate samples were collected at each sampling site. At each sampling interval, the sampler and stainless sieve were cleaned carefully with deionized water to reduce cross-contamination.

For the sediment sampling, the top sediment (0–2 cm) of lakeshore line was collected with a stainless shovel. Sediment samples were collected five times at each sampling site, about 5 m away from each other, and then mixed together and covered with aluminum foil. A 0.3 m × 0.2 m quadrat was used to delineate the scope of the sediment collection. An approximately 1 kg sediment sample was collected at each sampling site. Surface water and sediment samples were placed in sealed bags and placed in a 5 °C sampling box to avoid shaking and contamination during transportation. The samples then were transported to the laboratory for analysis as soon as possible. Although they were not the main subject of this study, macroplastics (>25 mm) and mesoplastics (5–25 mm) from both the lake centers and lakeshores were collected in the sampling process in order to help identify the types of plastics and the sources of debris plastics (Figure S1).

### 2.2. Isolation of Microplastics

In order to eliminate the interference of organisms and sediments in the collected samples, the microplastics need to be separated. Samples were pretreated using a method supported by the National Oceanic and Atmospheric Administration with some modifications [37]. Briefly, in treating the surface water samples, a hydrogen peroxide solution (30%, *v*/*v*) was used to remove visible organisms from the samples. Ferrous sulphate solution was used as catalyst. Density separation was carried out using a zinc chloride solution (1.5 g/cm^3^) to remove sands and minerals from samples. A simple density separator is used in this study [37]. An iron frame with an iron ring is placed on the laboratory bench, a glass funnel is fitted with a latex tubing on the bottom of the stem. A pinch clamp is settled to control the liquid flow from the glass funnel. The supernatants were collected in the density separator and then filtered through 0.22 μm pore size GF/C filter (Membrane Solutions LLC., Kent, WA, USA). Since natural air drying can curl the filter, all of the filters were dried in an oven set at 60°C.

Collected sediment samples were first treated by a freeze-drying method. Then 50 g (dw) sediment was transferred into a 1 L beaker, to which 400 mL of zinc chloride solution (1.5 g/cm^3^) and a stirring bar were added. After stirring for 30 minutes, samples were allowed to settle 24 h in order to achieve density separation. Supernatants then were collected and treated with surface water treatment method described above. Microplastics then were filtered onto the filters in the same manner as the surface water samples.

### 2.3. Observation and Identification of Microplastics

All of the treated filters were placed in a pre-cleaned Petri dish for observation. The Petri dishes were placed under a stereomicroscope with a digital camera (SZX7, Olympus, Tokyo, Japan) for examination. Suspected particles were identified according to their morphological characteristics. The identification was based on classification standards from previous studies [38,39,40]. The quantity, size, shape, and color of the microplastics were recorded. All of the microplastics then were divided into four categories according to their sizes: C1 (<0.5 mm), C2 (0.5–1 mm), C3 (1–3 mm) and C4 (3–5 mm). Using a classification system from previous studies, microplastics were divided into four categories according to their shapes: fiber, fragment, pellet and film [36,41]. Briefly, a long, thin line with a slender shape is called a fiber; fragments are hard piece of debris from a broken plastic item; debris with a thin layer is called film; and pellets are microplastics with spherical and cylindrical shapes. Although the method of microscopic identification has been used in many published studies, it is a relatively primary method of identification. A Renishaw inVia Raman spectroscope (Wotton-under-Edge, Gloucestershire, UK) was used for further identification. The wavelength of incident laser was set to 532 nm and the Raman spectra were from 50 to 3500 cm^−1^ [29,42].

### 2.4. Quality Assurance and Quality Control

A series of measures were adopted to avoid potential background contamination during sampling and laboratory processing. Researchers wore cotton lab coats and nitrile gloves during all of the processes. All of the containers and experimental instruments were pre-cleaned three times by ultrapure water and wrapped in aluminum foil when not in use. Ten blank tests using the methods described in Section 2.2 and Section 2.3 for determine the background values of contamination from the laboratory. Briefly, thirty liters distilled water was filtered onto a GF/C filter. Then these filters were kept in the lab for 72 h without any cover. After 72 h, these filters were observed by a stereomicroscope mentioned above. The results of blank tests shown that no microplastics were found in the filters. Absence of microplastic indicated that the background contamination in this study could be neglected.

## 3. Results

### 3.1. Abundances of Microplastics

Microplastics were found in all 50 surface water samples from 14 lakeshore sites and 22 lake center sites (Table 2). Because lakeshore sites are very different from the lake center sites, they are discussed separately in this study. The abundance of microplastics ranged from 616.67 to 2216.67 items/m^3^ and 716.67 to 2316.67 items/m^3^ in the lakeshore surface water of West Dongting Lake and South Dongting Lake, respectively. The highest concentrations of microplastics in both lakes were found at the outlets, site W04 and site S07. From the lakeshore surface water samples, it could be seen that the mean concentration of microplastics in South Dongting Lake was slightly higher than that in West Dongting Lake. Lake surface sites in the lake center sites show the converse situation. In the center of South Dongting Lake, the concentration of microplastics is slightly lower than in the center of West Dongting Lake. Concentration of microplastic in the wide South Dongting Lake has a microplastic abundance of 433.33 to 1500 items/m^3^, while the relatively narrow West Dongting Lake has a concentration from 366.67 to 1566.67 items/m^3^, respectively. In West Dongting Lake, sites on the eastern part of the lake showed a relatively higher concentration of microplastic than in the western part. South Dongting Lake showed a high concentration in the east and west and a low concentration in the center.

Microplastics were detected in all 14 sediment samples, as in the case of water samples. The abundance of microplastics in West Dongting Lake varied from 320 to 480 items/kg with the highest abundance detected in site W02, which is the entrance of the Songli Spillway. The abundances of microplastics in South Dongting Lake ranged from 200 to 1150 items/kg with the highest microplastic concentration found in site S03, an inflowing river site.

### 3.2. Shape, Color and Size of Microplastics

**Figure 2:**
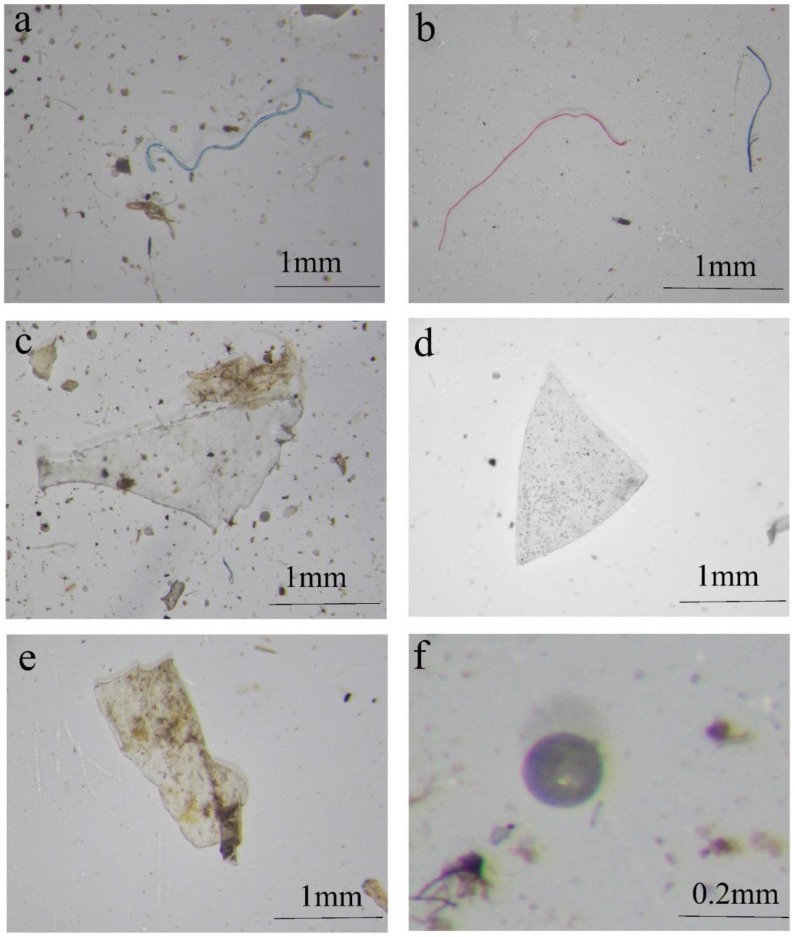
Typical photographs of microplastics: (**a**,**b**) fiber; (**c**,**d**) fragment; (**e**) film; (**f**) pellet.

**Figure 3:**
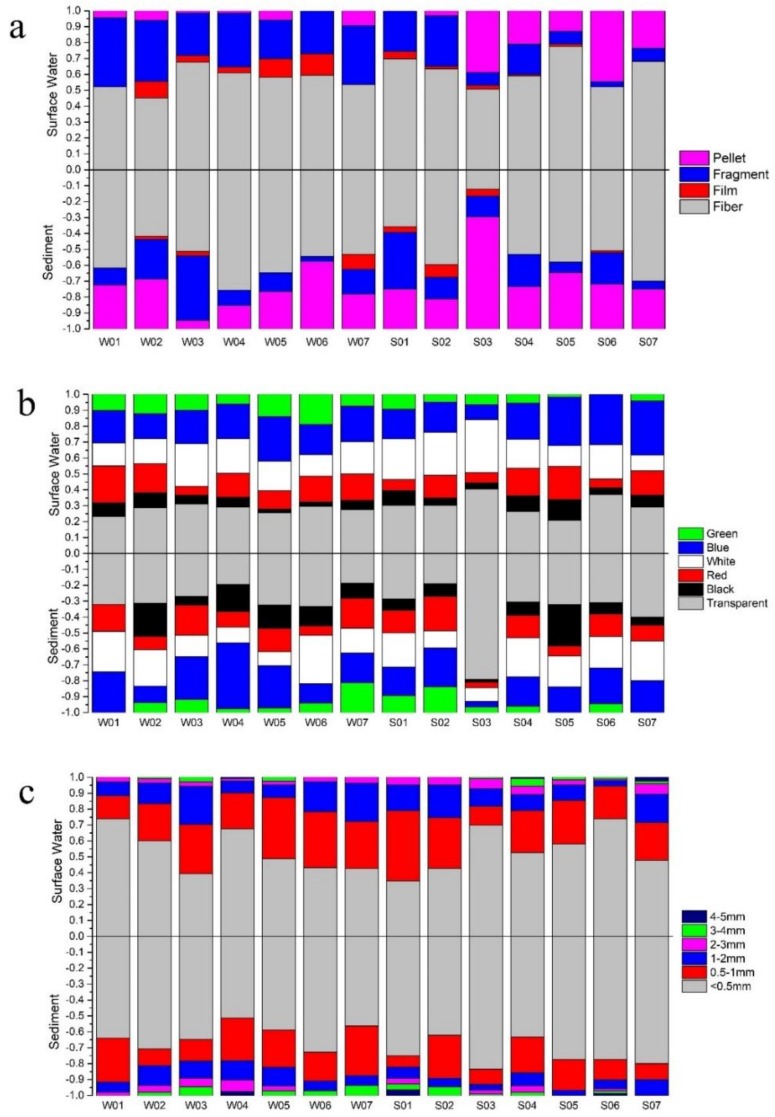
Proportion of (**a**) shape distribution (**b**) color distribution and (**c**) size distribution of microplastics in lakeshore sites.

**Figure 4:**
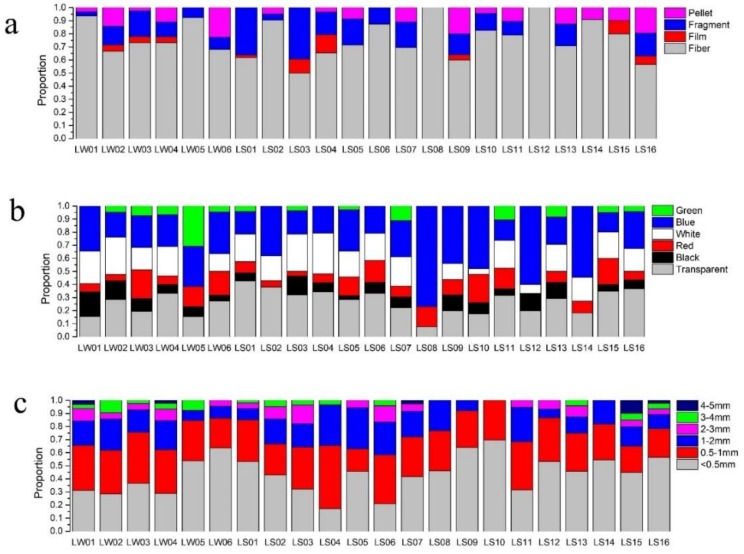
Proportion of (**a**) shape distribution (**b**) color distribution and (**c**) size distribution of microplastics in lake center sites.

Figure 2 show typical microplastic photographs. The results of the classification statistics are shown in Figure 3 and Figure 4. Collected microplastics were classified by three ways: shape, color, and size. Fibers were more common in most lakeshore surface water and sediment samples with a proportion of 12.17 to 77.42%. In sites central to the lakes, not only fibers were the largest in quantity, but there were even collected microplastics from two sites were all fibers. 

Transparent was the most dominant color across all samples, followed by white and blue. Red, black and green microplastics also were found in collected samples. In terms of size, small microplastics (<0.5 mm) were prevalent in most samples. The quantity of microplastics were correlated negatively with the size of microplastics (*p* < 0.05).

### 3.3. Composition of Microplastics

A total of 2393 microplastic particles were collected in this study. Since the study was limited by the cost and time of testing, not every microplastic particle was tested by micro-Raman spectroscopy. Therefore, in order to obtain a firm conclusion, we selected some representative microplastics for detection. This detection pattern has been used in published studies [17,29,43]. Those selected microplastics came from all sites, and they were in all shapes, sizes and colors. A total of 157 suspected microplastics were selected for micro-Raman spectroscopy identification. Fifty of them were from surface water samples and 107 were from sediment samples. 

The micro-Raman spectroscopy results are shown in Table 3. Various polymer types of microplastics were detected, including polyethylene terephthalate (PET), polystyrene (PS), polypropylene (PP), and polyethylene (PE). Polyvinyl chloride (PVC) microplastics were found only in sediment. PET dominated in sediment samples (50%) and PS dominated in surface water samples (40%). Of the particles, 7.01% of the particles were identified as non-plastic; theses were natural fibers or metals. PET dominated in sediment samples and PS dominated in surface water samples.

## 4. Discussion

### 4.1. Microplastic Pollution Level

There is no uniform standard for recording the abundance of microplastics in surface water and sediment, which means that not all of the results from published studies can be compared. Therefore, we selected published studies using the same statistical method as this study and compared the results in Tables S1 and S2. For surface water, the abundance of microplastics in West Dongting Lake and South Dongting Lake was similar to microplastics detected in Oujiang Estuary [44], Wu Lake [43], and Middle-Lower Yangtze River Basin [15]. Pollution level of West Dongting Lake and South Dongting Lake were slightly lower than that of East Dongting Lake located downstream [29]. This result is understandable given that East Dongting Lake is adjacent to Yueyang City, which has a denser population and more visitors. Compared with other lakes or rivers located in developed areas with dense population, such as Taihu Lake [45], Rhine River [46], and Hong Lake [29], relatively lower abundance of microplastics was found in the surface water of West Dongting Lake and South Dongting Lake. In contrast, Lake Chiusi and Lake Bolsena, which are located in sparsely populated areas, had a lower concentration of microplastic [47].

In terms of sediment, concentrations of microplastics were higher than in many rivers, lakes, estuaries and reservoirs [40,48,49], and are comparable to those detected in Beijiang River [50]. Higher pollution levels of microplastics still were being detected in some of the areas subject to intense human activity. There is an order of magnitude difference between the maximum concentrations of microplastic in these regions and in the areas examined, such as Lake Ontario [51]. Significantly, although the range of abundance can be comparable to those of rivers in urban areas, such as the Huangpu River, the average abundance is much lower than in those of rivers in urban areas [48]. Generally speaking, although the research areas do not have the most serious level of microplastic pollution, they still have higher concentrations than in most pristine areas. This fact indicates that microplastic pollution in the area still needs attention.

### 4.2. Source and Distribution of Microplastics

Plastic is an artificial product that is polymerized by addition polymerization or condensation polymerization. Any plastics, including microplastics, found in the environment are closely related to human activity. The highest microplastic concentrations in surface water were found in the outlets of West Dongting Lake and South Dongting Lake, with relatively low concentrations detected in sediment. This finding can be attributed to the increase in floating microplastics caused by the narrowing of the water surface, although the high current speed makes it difficult for microplastics to sink into the sediment. Compared with the vast lake surface, microplastics are more likely to stay on the shore under the effect of the current; this fact is similar to the fact that some coastal areas become converging sites of microplastics [39,52]. Hydrodynamic conditions have a significant effect on the distribution of microplastics. Yet hydrodynamic distribution of microplastics still needs further research [53]. 

Basing our approach on the information in existing studies and the situation of research areas, we suggest that riverine input is an important source for microplastics in West Dongting Lake and South Dongting Lake. The water in Dongting Lake mainly comes from inflowing rivers, and the most important four rivers were investigated in this study. The Xiangjiang River deserves our more attention. Xiangjiang is inflows from the eastern part of the South Dongting Lake. The Changsha-Zhuzhou-Xiangtan City Group located in the upstream of Xiangjiang River. More than 15 million people live in this area and its gross domestic product is as much as 1.54 trillion Yuan. A similar situation exists on the Zishui River, another important inflowing river. Developed industry and high levels of consumption may bring about a comfortable life there, but they also cause a large amount of microplastics to be discharged into the river. As important raw materials for the production of fabrics, plastic fibers of all colors are produced in large amounts [54,55]. Neither the environmental protection facilities of the factories nor wastewater treatment plants can thoroughly remove these fibers from the wastewater [56]. Much domestic sewage also goes into the water without any treatment. Characterized by large usage and easy breakage, fibers become the largest number of microplastics in the sample. This was reflected in this study, where a large number of fibers have been found in various sampling sites. In addition, large quantities of microplastics are produced by express delivery and takeout food, both of which have boomed in recent years. Plastics in disposable packaging plastics are discarded and broken into the transparent plastic fragments, which also were found in the sample [19]. Therefore, we suggested that rivers carrying domestic and industrial wastewater are the main sources of microplastics in the research areas. A similar situation has been found in Taihu Lake, the third largest freshwater lake in China. Therefore, on the basis of all of these findings, we suggest that rivers carrying domestic and industrial wastewater are the main sources of microplastics in the research areas [45,57].

Many published studies show that high concentrations of microplastic contamination are more likely to occur in developed areas with intensive human activity [24,43]. Yet there is no big city with a large population around the research areas. Miluo and Yuanjiang are located on the east and south sides of the research area, respectively. The populations of these two counties add up to less than 2 million people, which is not a large population compared to the rest of China. However, there are not only residents in this area, but also a large number of tourists. Dongting Lake has not only beautiful natural scenery, but also many famous scenic spots with profound cultural significance. According to the forecasts of the local tourism companies, it is expected that by 2020, more than 300 million tourists will visit here each year. This trend is reflected in the distribution of microplastics in the research areas, where tourist attractions and hotels in the east and south show a higher concentration of microplastics than in the west and north. Moreover, in order to develop tourism, a series of supporting facilities have been built along Dongting Lake, the most important of which is the lakeside road. These developments have led to a significant increase in traffic. Most of the industries originally distributed along the lake have been closed or relocated in recent years. At present, the industries located in the lakeside area primarily include dozens of shipbuilding enterprises, which mainly produce yachts; more than a thousand ships have been produced there each year. In addition to plastic waste discarded by tourists, tire wear from vehicles and paint spilling from ships are all sources of microplastics.

In addition to these tourist and industrial sites, some villages are scattered along the lakeside. The inhabitants of these villages live by planting, breeding animals and fishing. Some of the transparent and green fibers found in the samples belong to fishing tools, such as fishing nets. Besides, some of the thinnest transparent films are likely to be formed by the breaking of agricultural mulch. Despite the wide range of uses for agricultural mulch, such as insulation, moisturizing, insect and disease prevention, the characteristics of easily broken and difficult to recover have led to controversy in China. In addition, simple living habits and poor drainage systems have led to microbeads in personal care products entering the environment. These microbeads are the source of the pellets in the sample. As a freshwater lake, center of the lake will not become a sink for the accumulation of microplastics as the ocean and saltwater lake have become. On the contrary, this study found that the concentration of microplastics in the sparsely populated central area of the lake was significantly lower than those in oceans and in saltwater lakes, due to the effects of the current [17,58,59].

### 4.3. Composition of Microplastics

According to the composition of the microplastics under study (Table 3), a total of five types of plastics were found in the sample. As the largest number of microplastic type found in this study, PET can be spun and reprocessed to make an important synthetic fiber: Dacron. Dacron has high strength, good elasticity, and strong heat resistance, and thermoplasticity, all of which make it useful in the garment, construction, and automobile industries. According to local intra industry statistics, these and other products containing PET account for more than 80% of the clothing market. The results of this study indicate that textile is the main source of fiber. PE is important raw material for fishing nets, and they are also one of the sources of fiber. In this study, PE dominated in fragments, followed by PS and PP. PE, PS and PP are important material of disposable plastic containers for carried out foods. Moreover, PS is also used to make microbeads in personal care products and cosmetics [60]. While these findings reflect the living conditions and habits of lakeside residents, we suggest these fragments come in even greater amounts from the rubbish discarded by tourists. Several microplastics were also identified as PVC, a type of plastic found only in sediment samples. Although the number of positive PVC samples was only 4.46% of all identified particles, it deserves more attention. PVC can be prepared by a substitution reaction of ethylene, chlorine and catalyst. Due to its resistance to fire and heat, PVC is widely used in a variety of industries and products. Since its industrial production in the 1930s, PVC has been widely used in construction; in the production of electronics, furniture, toys, clothing, packaging and even healthcare. PVC may leach harmful plasticizers, antiaging agents, and other elements, but PVC also is the main source of dioxins, which are highly toxic to aquatic organisms and ultimately affect humans in the food chain. Research on microplastics, being an emerging pollutant, still has many deficiencies. As the largest producer and user of plastics in the world, China has carried out a large number of studies on microplastics and these studies, in combination with those carried out in other countries, yield a great deal of valuable baseline data [7,61]. However, most of these studies focus on abundance, distribution, morphological features, and composition since the pollution levels of microplastics are determined only by abundance or the weight of collected particles. However, there are many types of plastic and the ecological risks of various types of plastics significantly differ. Treating different chemical compositions of microplastics with the same method may adversely affect the formulation of relevant policies. The ecological risk assessment model of microplastics has been established on the basis of chemical toxicity and thermodynamics of plastics [48,62].However, this is only a very preliminary attempt. In the establishment and optimization of ecological risk assessment model of microplastics, researchers need to make more efforts.

## 5. Conclusions

The surface water and sediment in West Dongting Lake and South Dongting Lake are generally polluted by microplastics. Various shapes, colors, sizes, and chemical compositions of microplastics were found in these research areas. The levels of microplastic pollution found in these two vast freshwater lakes are currently at moderate levels. The distribution and morphological features of the detected microplastics indicate that the inflowing rivers are important sources of microplastics in the research areas. Human activity in the areas under study is another important source of microplastics. Finally, hydrodynamic conditions significantly affect the distribution of microplastics. In addition to abundance, microplastics with different polymer composition should also be considered. Further work should be carried out to explore the effect of hydrodynamic conditions on the distribution of microplastics and establishment of a reasonable and complete ecological risk assessment model.

## Figures and Tables

**Table 1:** Information of each lakeshore sites in this study.

Site	Place	Describe	Latitude	Longitude
W01	Shahekou	Lishui River	29°10’41”	112°12’21”
W02	Nanzui	Entrance of Songli Spillway	29°3’32”	112°18’13”
W03	Dongnan Lake	Resort	28°53’28”	112°19’49”
W04	Xiaohezui	Outlet of West Dongting Lake	29°50’38”	112°19’14”
W05	Jiangjiazui	State controlled section	28°49’42”	112°11’37”
W06	Potou	Yuanjiang River	28°54’8”	112°6’14”
W07	Niujiaojian	Village	28°47’53”	112°26’37”
S01	Liaodaokou	Eyot	28°51’37”	112°33’38”
S02	Wanzihu Village	State controlled section	28°47’53”	112°26’37”
S03	Wanjiazui	Zishui River	28°36’15”	112°21’46”
S04	Zhangshugang	Xiangjiang River	28°33’40”	112°48’19”
S05	Fengshutang	Village	28°48’57”	112°38’19”
S06	Yugong Temple	Inflowing site of Xiangjiang River and Zishui River	28°49’41”	112°53’44”
S07	Lujiao	Outlet of South Dongting Lake	29°9’29”	113°0’0”

**Table 2:** Abundance of microplastics collected from surface water and sediment.

Sample	Sites	Mean Abundance	Range of Abundance
Lakeshore Surface water (items/m3)	West Dongting Lake	1345.24 ± 560.81	616.67–2216.67
	South Dongting Lake	1464.29 ± 559.05	716.67–2316.67
Lakeshore Sediment (items/kg)	West Dongting Lake	388.57 ± 66.19	320–480
	South Dongting Lake	501.43 ± 331.18	200–1150
Lake center Surface water (items/m3)	West Dongting Lake	966.67 ± 415.8	433.33–1500
	South Dongting Lake	866.67 ± 352.56	366.67–1566.67

**Table 3:** Polymer types for the result of the micro-Raman spectroscopy.

Types	Surface Water	Sediment	Total	Percentage (%)
PET	14	49	63	40.13
PP	8	10	18	11.46
PS	19	14	33	21.02
PE	6	19	25	15.92
PVC	0	7	7	4.46
Non-plastic	3	8	11	7.01
Plastic	47	99	146	92.99
Total	50	107	157	100.00

## Supplementary Materials

The following are available online at http://www.mdpi.com/1660-4601/15/10/2164/s1, Figure S1: Typical macroplastics and mesoplatics collected in this study, Table S1: Abundance comparison with other freshwater areas (Surface water)., Table S2: Abundance comparison with other freshwater areas (Sediment).
