# Supplementary material for: Microplastics in Sediment and Surface Water of West Dongting Lake and South Dongting Lake: Abundance, Source and Composition
Source: Int J Environ Res Public Health. 2018 Oct 1;15(10):2164. doi: 10.3390/ijerph15102164 (PMC6210014; doi:10.3390/ijerph15102164)
Supplement: Supplementary file 1 [file ijerph-15-02164-s001.pdf]

International Journal of
Environmental Research and Public Health

Supplementary Material:

Figure S1. Typical marcoplastics and mesoplatics collected in this study.

Table S1. Abundance comparison with other freshwater areas (Surface water).

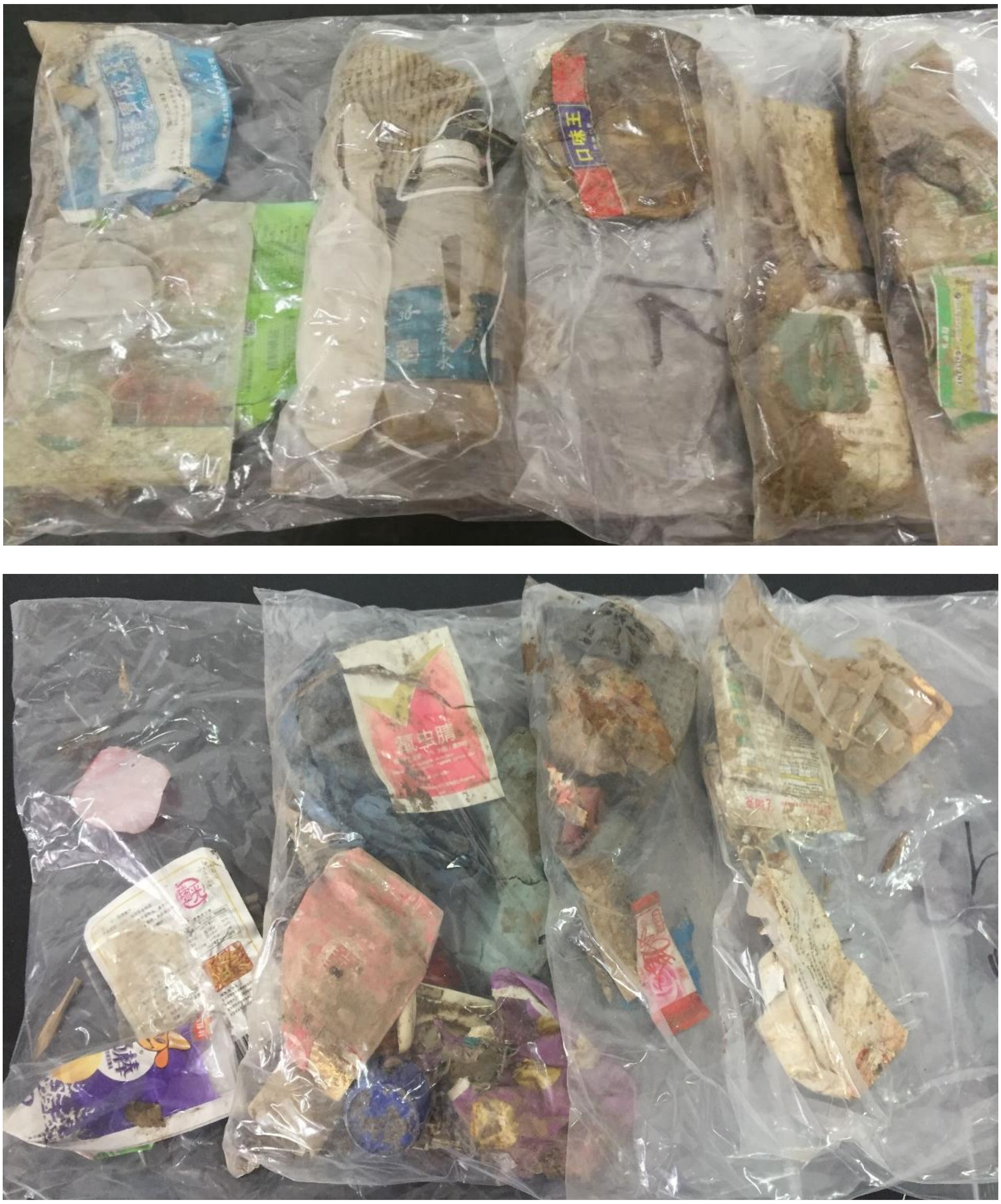

Figure S1. Typical marcoplastics and mesoplatics collected in this study.

Table S1. Abundance comparison with other freshwater areas (Surface water).

Surface Water	Location	Abundance (Items/m^3)	Reference
Los Angeles River	USA	22–12,932	[1]
Tamar Estuary	UK	0.028	[2]
Yangtze River Estuary	China	500–10,200	[3]
Oujiang Estuary	China	100–4100	[4]
Seine River	France	3–108	[5]
Great Lakes tributaries	North America	0.05–32	[6]
Lake Bolsena	Italy	0.82–4.42	[7]
Lake Chiusi	Italy	2.68–3.36	[7]
Taihu Lake	China	3400–25,800	[8]
Bei Lake	China	7800–10,050	[9]
Wu Lake	China	1160–2380	[10]
Three Gorges Dam	China	1594–12,611	[11]
Rhine River and Meuse River	Netherlands and Germany	100–18,700	[12]
Middle-Lower Yangtze River Basin	China	500–3100	[13]
Antua River	Portugal	58–1265	[14]
Hong Lake	China	1250–4650	[15]
East Dongting Lake	China	900–2800	[15]
West Dongting Lake	China	433–2217	This study
South Dongting Lake	China	367–2316	This study

Table S2. Abundance comparison with other freshwater areas (Sediment).

Sediment	Location	Abundance (Items/kg)	Reference
Lagoon of Venice	Italy	672–2175	[16]
Rhine River	Germany	228–3763	[17]
Taihu Lake	China	11–234.6	[8]
Lake Chiusi	Italy	205–266	[7]
Lake Ontario	Canada	20–27,830	[18]
Beijiang River	China	178–544	[9]
Huangpu River	China	410–1600	[19]
Edgbaston Pool	UK	250–300	[20]
Yangtze River Esuary	China	20–340	[21]
River Thames Tributary	UK	18.5–66	[22]
Antua River	Portugal	18–619	[14]
Three Gorges Dam	China	25–300	[11]
West Dongting Lake	China	320–480	This study
South Dongting Lake	China	200–1150	This study

References

1. Moore, C.J.; Lattin, G.L.; Zellers, A. Quantity and type of plastic debris flowing from two urban rivers to coastal waters and beaches of Southern California. Journal of Integrated Coastal Zone Management 2011; 11: 65-73.
2. Sadri SS, Thompson RC. On the quantity and composition of floating plastic debris entering and leaving the Tamar Estuary, Southwest England. Marine Pollution Bulletin 2014; 81: 55-60.
3. Zhao S, Zhu L, Wang T, et al. Suspended microplastics in the surface water of the Yangtze Estuary System, China: First observations on occurrence, distribution. Marine Pollution Bulletin 2014; 86: 562-568.
4. Zhao S, Zhu L, Li D. Microplastic in three urban estuaries, China. Environmental Pollution 2015; 206: 597-604.
5. Dris R, Gasperi J, Rocher V, et al. Microplastic contamination in an urban area: a case study in Greater Paris. Environmental Chemistry 2015; 12: 592.
6. Baldwin AK, Corsi SR, Mason SA. Plastic Debris in 29 Great Lakes Tributaries: Relations to Watershed Attributes and Hydrology. Environmental Science & Technology 2016; 50: 10377-10385.
7. Fischer EK, Paglialonga L, Czech E, et al. Microplastic pollution in lakes and lake shoreline sediments-A case study on Lake Bolsena and Lake Chiusi (central Italy). Environmental Pollution 2016; 213: 648-657.
8. Su L, Xue Y, Li L, et al. Microplastics in Taihu Lake, China. Environmental Pollution 2016; 216: 711-719.
9. Wang J, Peng J, Tan Z, et al. Microplastics in the surface sediments from the Beijiang River littoral zone: Composition, abundance, surface textures and interaction with heavy metals. Chemosphere 2017; 171: 248-258.
10. Wang W, Ndungu AW, Li Z, et al. Microplastics pollution in inland freshwaters of China: A case study in urban surface waters of Wuhan, China. Science of The Total Environment 2017; 575: 1369-1374.
11. Di M, Wang J. Microplastics in surface waters and sediments of the Three Gorges Reservoir, China. Science of The Total Environment 2018; 616-617: 1620-1627.
12. Leslie HA, Brandsma SH, van Velzen MJM, et al. Microplastics en route: Field measurements in the Dutch river delta and Amsterdam canals, wastewater treatment plants, North Sea sediments and biota. Environment International 2017; 101: 133-142.
13. Su L, Cai H, Kolandhasamy P, et al. Using the Asian clam as an indicator of microplastic pollution in freshwater ecosystems. Environmental Pollution 2018; 234: 347-355.
14. Rodrigues MO, Abrantes N, Gonçalves FJM, et al. Spatial and temporal distribution of microplastics in water and sediments of a freshwater system (Antuã River, Portugal). Science of The Total Environment 2018; 633: 1549-1559.
15. Wang W, Yuan W, Chen Y, et al. Microplastics in surface waters of Dongting Lake and Hong Lake, China. Science of The Total Environment 2018; 633: 539-545.
16. Vianello A, Boldrin A, Guerriero P, et al. Microplastic particles in sediments of Lagoon of Venice, Italy: First observations on occurrence, spatial patterns and identification. Estuarine, Coastal and Shelf Science 2013; 130: 54-61.
17. Klein S, Worch E, Knepper TP. Occurrence and Spatial Distribution of Microplastics in River Shore Sediments of the Rhine-Main Area in Germany. Environmental Science & Technology 2015; 49: 6070-6076.
18. Ballent A, Corcoran PL, Madden O, et al. Sources and sinks of microplastics in Canadian Lake Ontario nearshore, tributary and beach sediments. Marine Pollution Bulletin 2016; 110: 383-395.
19. Peng G, Xu P, Zhu B, et al. Microplastics in freshwater river sediments in Shanghai, China: A case study of risk assessment in mega-cities. Environmental Pollution 2018; 234: 448-456.
20. Vaughan R, Turner SD, Rose NL. Microplastics in the sediments of a UK urban lake. Environmental Pollution 2017; 229: 10-18.
21. Peng G, Zhu B, Yang D, et al. Microplastics in sediments of the Changjiang Estuary, China. Environmental Pollution 2017; 225: 283-290.
22. Horton AA, Svendsen C, Williams RJ, et al. Large microplastic particles in sediments of tributaries of the River Thames, UK – Abundance, sources and methods for effective quantification. Marine Pollution Bulletin 2017; 114: 218-226.
